# Development and External Validation of Machine Learning Models for Diabetic Microvascular Complications: Cross-Sectional Study With Metabolites

**DOI:** 10.2196/41065

**Published:** 2024-03-28

**Authors:** Feng He, Clarissa Ng Yin Ling, Simon Nusinovici, Ching-Yu Cheng, Tien Yin Wong, Jialiang Li, Charumathi Sabanayagam

**Affiliations:** 1 Singapore Eye Research Institute Singapore National Eye Centre Singapore Singapore; 2 Department of Statistics and Data Science National University of Singapore Singapore Singapore; 3 Ophthalmology and Visual Sciences Academic Clinical Program Duke-NUS Medical School Singapore Singapore

**Keywords:** machine learning, diabetic microvascular complication, diabetic kidney disease, diabetic retinopathy, biomarkers, metabolomics, complication, adult, cardiovascular disease, metabolites, biomedical big data, kidney disease

## Abstract

**Background:**

Diabetic kidney disease (DKD) and diabetic retinopathy (DR) are major diabetic microvascular complications, contributing significantly to morbidity, disability, and mortality worldwide. The kidney and the eye, having similar microvascular structures and physiological and pathogenic features, may experience similar metabolic changes in diabetes.

**Objective:**

This study aimed to use machine learning (ML) methods integrated with metabolic data to identify biomarkers associated with DKD and DR in a multiethnic Asian population with diabetes, as well as to improve the performance of DKD and DR detection models beyond traditional risk factors.

**Methods:**

We used ML algorithms (logistic regression [LR] with Least Absolute Shrinkage and Selection Operator and gradient-boosting decision tree) to analyze 2772 adults with diabetes from the Singapore Epidemiology of Eye Diseases study, a population-based cross-sectional study conducted in Singapore (2004-2011). From 220 circulating metabolites and 19 risk factors, we selected the most important variables associated with DKD (defined as an estimated glomerular filtration rate <60 mL/min/1.73 m^2^) and DR (defined as an Early Treatment Diabetic Retinopathy Study severity level ≥20). DKD and DR detection models were developed based on the variable selection results and externally validated on a sample of 5843 participants with diabetes from the UK biobank (2007-2010). Machine-learned model performance (area under the receiver operating characteristic curve [AUC] with 95% CI, sensitivity, and specificity) was compared to that of traditional LR adjusted for age, sex, diabetes duration, hemoglobin A_1c_, systolic blood pressure, and BMI.

**Results:**

Singapore Epidemiology of Eye Diseases participants had a median age of 61.7 (IQR 53.5-69.4) years, with 49.1% (1361/2772) being women, 20.2% (555/2753) having DKD, and 25.4% (685/2693) having DR. UK biobank participants had a median age of 61.0 (IQR 55.0-65.0) years, with 35.8% (2090/5843) being women, 6.7% (374/5570) having DKD, and 6.1% (355/5843) having DR. The ML algorithms identified diabetes duration, insulin usage, age, and tyrosine as the most important factors of both DKD and DR. DKD was additionally associated with cardiovascular disease history, antihypertensive medication use, and 3 metabolites (lactate, citrate, and cholesterol esters to total lipids ratio in intermediate-density lipoprotein), while DR was additionally associated with hemoglobin A_1c_, blood glucose, pulse pressure, and alanine. Machine-learned models for DKD and DR detection outperformed traditional LR models in both internal (AUC 0.838 vs 0.743 for DKD and 0.790 vs 0.764 for DR) and external validation (AUC 0.791 vs 0.691 for DKD and 0.778 vs 0.760 for DR).

**Conclusions:**

This study highlighted diabetes duration, insulin usage, age, and circulating tyrosine as important factors in detecting DKD and DR. The integration of ML with biomedical big data enables biomarker discovery and improves disease detection beyond traditional risk factors.

## Introduction

Diabetes is a complex metabolic disorder and a major global health problem of our time. In 2021, it affected 536.6 million adults worldwide, with a projected surge to 783.2 million by 2045 [1]. With the rapidly growing population with diabetes and the greater longevity over time, the burden of associated complications is expected to increase in parallel [2]. Among these, diabetic kidney disease (DKD) was estimated to develop in around 40% of the population with diabetes [3], while diabetic retinopathy (DR) would manifest in approximately 35% [4]. Left undetected and untreated, these microvascular complications could substantially elevate the risk of cardiovascular disease (CVD), end-stage renal disease, and permanent vision loss, resulting in compromised quality of life and shortened life expectancy [2,5,6]. Yet, timely and accurate diagnosis of DKD and DR remains a challenge because of their asymptomatic progression in the early stages [7]. Although factors such as age, sex, diabetes duration, hemoglobin A_1c_ (HbA_1c_) %, systolic blood pressure (SBP), and BMI have been identified as the risk contributors for DKD and DR, they offer only partial insights into the variability of risk among individuals.

To enable risk prediction and exploration of the underlying metabolic pathways, metabolomics has been increasingly used for biomarker discovery in diabetes and its complications [7-10]. Abnormalities in amino acids and lipids metabolism have been linked to both DKD and DR [7]. Given the similar microvascular structure, physiology, and pathogenic features of the kidney and the eye, these common metabolic traits may indicate biochemical pathways shared by the 2 complications, or both being manifestations of a latent systematic condition [11]. However, to the best of our knowledge, investigations into the commonalities and differences between DKD and DR in terms of their metabolic basis have been somewhat scarce [7,12]. Moreover, prior research was often limited by inadequate sample sizes, lack of replication, and restricted data analysis methods [13].

In this study, we aimed to fill these gaps by analyzing 220 circulating metabolites and 19 established risk factors as predictors of prevalent DKD and DR in a retrospective Asian adult population with diabetes. We used 2 machine learning (ML) algorithms, logistic regression (LR) with Least Absolute Shrinkage and Selection Operator (LASSO [14]), and a gradient-boosting decision tree (GBDT [15]). Compared with traditional statistical methods, these ML algorithms excel in handling high-dimensional data with complex relationships and can quantify the relative contribution of individual variables through variable importance scores. Based on these scores, we selected the top variables to develop ML models for DKD and DR detection and validated them externally in UK biobank (UKBB [16]) data. Additionally, we developed reference models using LR adjusted for traditional risk factors. All models were evaluated using the area under the receiver operating characteristic curve (AUC) with 95% CI, sensitivity, and specificity. Finally, we discussed the potential role of the selected metabolites in DKD and DR with reference to previous studies.

## Methods

### Data Sets

We derived this study’s data from the Singapore Epidemiology of Eye Diseases study (SEED [17]), a population-based cross-sectional study conducted in Singapore from 2004 to 2011. Detailed methodology has been reported elsewhere [17]. In brief, we recruited 10,033 adults aged 40-80 years using age-stratified random sampling. Participants completed interviewer-administered questionnaires, underwent ocular examinations, and provided samples for biochemical laboratory tests. The cohort included 3280 Malay (2004-2006, response rate 78.7%), 3400 Indian (2007-2009, response rate 75.6%), and 3353 Chinese (2009-2011, response rate 72.8%) individuals.

For external validation, we used data from UKBB, an open-access resource of prospective data set collected in the UK from 2007 to 2010, with over 500,000 participants recruited between the ages of 40 to 69 [16].

### Ethical Considerations

Both SEED and UKBB studies were conducted per the Declaration of Helsinki. Ethical approval was obtained from the SingHealth Institutional Review Board for SEED (2018/2717, 2018/2921, 2018/2006, 2018/2594, 2018/2570, 2015/2279, and 2012/487/A) and from North West Multi-centre Research Ethics Committee for UKBB (21/NW/0157). Written informed consent was provided by all participants during the primary data collection. Due to the retrospective nature of our study and the use of deidentified health information, the SingHealth Institutional Review Board approved this study without requiring additional patient consent.

### Definition of Outcome and Variables

In SEED, diabetes was defined as meeting any of the following criteria: HbA_1c_% >6.5, random blood glucose >11.1 mmol/L, self-reported physician-diagnosed diabetes, or the use of antidiabetic medication including insulin. In UKBB, we applied the same definition but additionally included individuals with DR if the aforementioned variables were missing.

DKD was defined as an estimated glomerular filtration rate (eGFR) <60 mL/min/1.73m^2^ in people with diabetes. The eGFR values were calculated from blood creatinine concentrations using the chronic kidney disease epidemiology collaboration equation [18].

For SEED participants with diabetes, DR severity in each eye was graded from fundus photographs by certified ophthalmic graders according to the standard protocol of Early Treatment Diabetic Retinopathy Study (ETDRS) [2,19] with 6 stages: no DR (level 10-20), minimal (level 20), mild (level 35), moderate (levels 43 to 47), severe (level 53), and proliferative DR (levels >60). We defined the DR outcome as having an ETDRS level ≥20 in at least one eye (ie, any DR). In UKBB, DR severity was not graded. Therefore, we identified DR cases based on *the International Classification of Diseases, Tenth Revision (ICD-10)* code “H36.0” in their health-related outcomes (UKBB Data-Field: 41270) [16]. Supplementary analysis in SEED also considered moderate or worse DR (level >43 in at least one eye) as an additional outcome.

We considered a total of 239 variables for biomarker selection (Multimedia Appendix 1). Among these, 19 variables were identified through a literature review, comprising 6 traditional risk factors (age, sex, duration of diabetes, HbA_1c_%, SBP, and BMI), and 13 extended risk factors related to lifestyle (alcohol consumption and smoking), medication use (insulin, anticholesterol, and antihypertensive medication), clinic or biochemistry (diastolic blood pressure [DBP], pulse pressure [PP], random blood glucose, total cholesterol, high-density lipoprotein [HDL] cholesterol, and low-density lipoprotein cholesterol), and comorbidity conditions (hypertension and history of CVD). Hypertension in both cohorts was defined as self-reported physician-diagnosed hypertension, SBP >140 mm Hg, DBP >80 mm Hg, or the use of antihypertensive medication.

Using nuclear magnetic resonance techniques (Nightingale Health), we quantified the concentration of 228 circulating metabolites from patients’ blood samples. Of these, glycerol, pyruvate, and glutamine were not available for Malay individuals, creatinine was used in eGFR calculation and DKD outcome definition, while 4 metabolites (total, HDL, low-density lipoprotein cholesterols, and random blood glucose) were duplicated with those measured in biochemistry tests. Therefore, these variables were excluded from the analysis, leaving us with 220 metabolites from 15 categories (amino acids, apolipoproteins, cholesterol, cholesterol esters, fatty acids, fluid balance, free cholesterols, glycolysis-related metabolites, inflammation, ketone bodies, triglycerides, lipoprotein particle sizes, lipoprotein subclasses, lipoprotein lipid ratios, and other lipids).

### Statistical Analysis

#### Inclusion and Exclusion Criteria

**Figure 1:**
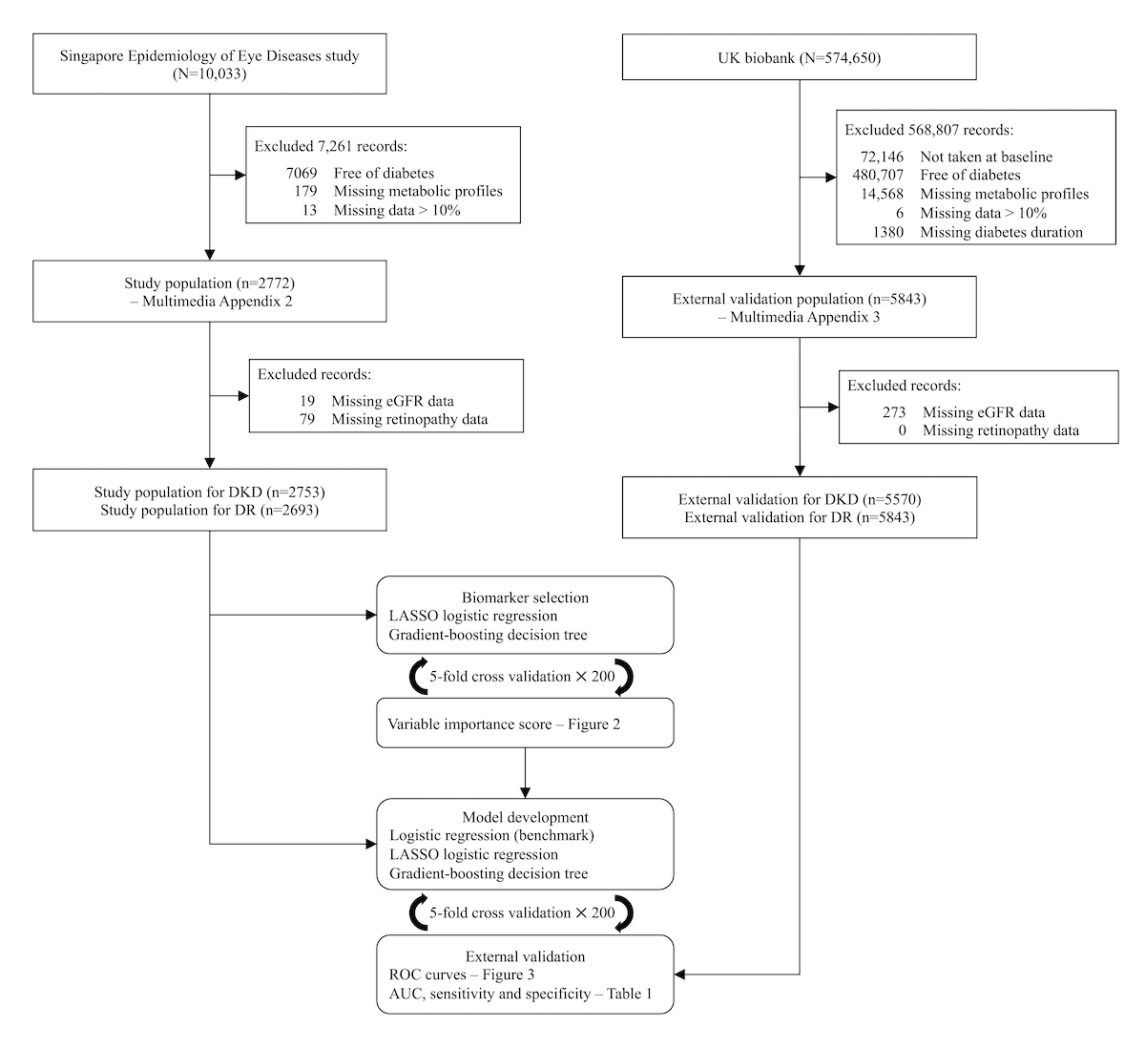
Exclusion criteria and machine learning workflow. AUC: area under the receiver operating characteristic curve; DKD: diabetic kidney disease; DR: diabetic retinopathy; eGFR: estimated glomerular filtration rate; LASSO: Least Absolute Shrinkage and Selection Operator; ROC: receiver operating characteristic.

From the initial SEED data set of 10,033 participants, we excluded those free of diabetes (n=7069), missing metabolomics profiles (n=179), or missing more than 10% (n=13) of the data, to obtain a final study population of 2772 individuals. Similarly, we identified 5843 UKBB participants eligible for external validation after data cleaning (Figure 1).

#### Descriptive Statistics

We categorized SEED participants into 4 groups based on their DKD and DR status, and summarized group characteristics as number (%), mean (SD), or median (IQR) as appropriate for each variable. Differences among groups were assessed using 2-sided *P* values derived from Pearson *χ*^2^ tests or Fisher exact test for categorical variables, and Kruskal-Wallis rank sum tests for numeric variables. We also evaluated the interpopulation differences between SEED and UKBB using Pearson *χ*^2^ tests and Mann-Whitney *U* tests as appropriate for each variable. Some subcategories may not add up due to the presence of missing data.

#### ML Algorithms

We used LASSO [14] and GBDT [15] to identify the key biomarkers and develop disease detection models for DR and DKD. LASSO is an extension of traditional LR that does not require the independence of variables. Therefore, LASSO is suitable for high-dimensional data sets where issues such as multicollinearity often arise. During parameter optimization, LASSO automatically shrinks the coefficients of the less-important variables to zero, while retaining nonzero coefficients for the important ones to achieve biomarker selection. Its strengths include relatively straightforward computation and parameter tuning compared to other ML algorithms. However, its scope is limited to examining only the linear associations between continuous variables and the log-odds. To account for possible nonlinear effects and variable interactions, we additionally implemented the GBDT algorithm, which constructs a sequence of interdependent decision trees to collectively make a decision. During the process, the algorithm assesses each variable’s contribution to minimizing prediction errors, returning a score for its relative influence. We then applied a predefined threshold to select the most influential variables for disease detection based on their scores in each selection round. GBDT is known for its adaptability to various data distributions with generally strong performances, yet its hyper-parameter tuning and computation can be rather time-consuming, with a higher chance of overfitting, and less transparency compared to LASSO.

#### Model Development and Validation

In SEED, we handled missing data by excluding variables and participants with high levels of missing information, yielding a final data set with each variable containing less than 6% (161/2772) missing data and a participant-level missing data percentage of 10% or less (23/239, Multimedia Appendix 2). Following this, missing data imputation was carried out by using mean values for numeric variables, and modes for categorical variables as appropriate. To mitigate selection bias arising from data division into training and validation sets, we averaged the results over 200 random repeats of 5-fold cross-validation. In each repeat, the imputed SEED data set was randomly divided into 5 subsets (ie, folds) of equal sample size by stratified sampling to ensure consistent case rates. Each fold (553/2772, 20% of data) took its turn as the validation set, while the remaining 4 folds (2219/2772, 80% of data) were used for model training and variable selection. From 200 replicates we generated 1000 sets of variable selection results, based on which we quantified each variable’s contribution to the model’s performance by calculating a variable importance score, as the variable’s selection frequency during the repeated cross-validation process.

Next, we arranged the variables in descending order of their selection frequencies and included only those selected 990 times or more (ie, selection frequency 0.990) in the final model. To evaluate the performance of these new models, we performed another 200 random repeats of 5-fold cross-validation but used only the complete cases (ie, no missing data imputation). The final machine-learned models were compared with the multivariate LR models adjusted for the 6 established risk factors including age, sex, diabetes duration, HbA_1c_%, SBP, and BMI. The performance of these models was evaluated in internal and external validation using AUC with 95% CI, sensitivity, and specificity. We reported sensitivity and specificity at the optimal threshold, where sensitivity equals specificity. Additionally, in the context of DR and DKD detection, where the cost of false negatives is generally higher than that of false positives, we prioritized higher sensitivity over higher specificity by setting the probability threshold at 0.8 sensitivity to compare the specificity. The AUC difference between machine-learned models and the traditional model was evaluated using the test by DeLong et al [20]. For the supplementary analysis, we examined the importance of the top machine-learned variables within nested models, to check for any marginal increase in AUC with additional variables. We also calculated the AUC for ML models with all the variables.

#### Metabolites Selection

Finally, we focused on the metabolites that were consistently selected by both ML algorithms. We quantified their associations with DKD and DR respectively, using odds ratios (OR) per SD increment with 95% CI, and *P* values from multivariate LR models adjusted for age, sex, diabetes duration, insulin use, HbA_1c_%, PP, BMI, cholesterol, and HDL cholesterol.

We conducted all the analyses in R version 4.0.2. (R Foundation) and defined statistical significance as *P*<.05.

## Results

### Population Characteristics

Figure 1 illustrates the data cleaning and analysis workflow. In the SEED population with diabetes, 2674 people had information on both disease outcomes (Multimedia Appendix 3). Of these, 1657 (62%) had neither DKD nor DR, 338 (12.6%) had DKD but not DR, 496 (18.5%) had DR but not DKD, and 183 (6.8%) had both DKD and DR. People with DKD, regardless of their DR status, tended to be older, with higher PP, higher SBP, and lower DBP. They also had higher levels of HDL cholesterol, more history of hypertension and CVD, and were more likely to have used anticholesterol medication and antihypertensive medication. However, they had a lower smoking rate and reported less alcohol consumption than those without DKD. People with DR, regardless of their DKD status, tended to have a longer duration of diabetes, with the use of antidiabetic medication and insulin, higher HbA_1c_%, and random blood glucose levels. They also had a lower BMI and lower total cholesterol levels. For people with both complications, the abovementioned characteristics further differed from those with neither complication. However, in terms of sex distribution, no significant difference was observed (*P*=.09).

We also found differences between SEED and UKBB in terms of demographics, lifestyle factors, biochemical laboratory results, and medical history (Multimedia Appendix 4). In particular, UKBB participants had a lower prevalence of both DKD (374/5570, 6.7%) and DR (355/5843, 6.1%) as compared to SEED (DKD prevalence: 555/2753, 20.2%, and DR prevalence: 685/2693, 25.4%), with only 0.7% (41/5570) having both complications. SEED had a median age of 61.7 (IQR 53.5-69.4) years and 49.1% (1361/2772) women, whereas UKBB had a median age of 61.0 (IQR 55.0-65.0) years and 35.8% (2090/5843) women. SEED was a multiethnic sample based in Singapore with 36.8% (1020/2772) Malay, 45.5% (1262/2772) Indian, and 17.7% (490/2772) Chinese individuals. In contrast, over 81.8% (4778/5843) of the UKBB participants were British, with the rest being Indian (217/5843, 3.7%), Irish (132/5843, 2.3%), Caribbean (93/5843, 1.6%), African (85/5843, 1.5%), or other ethnicities (538/5843, 9.2%).

### Model Development and Validation

**Figure 2:**
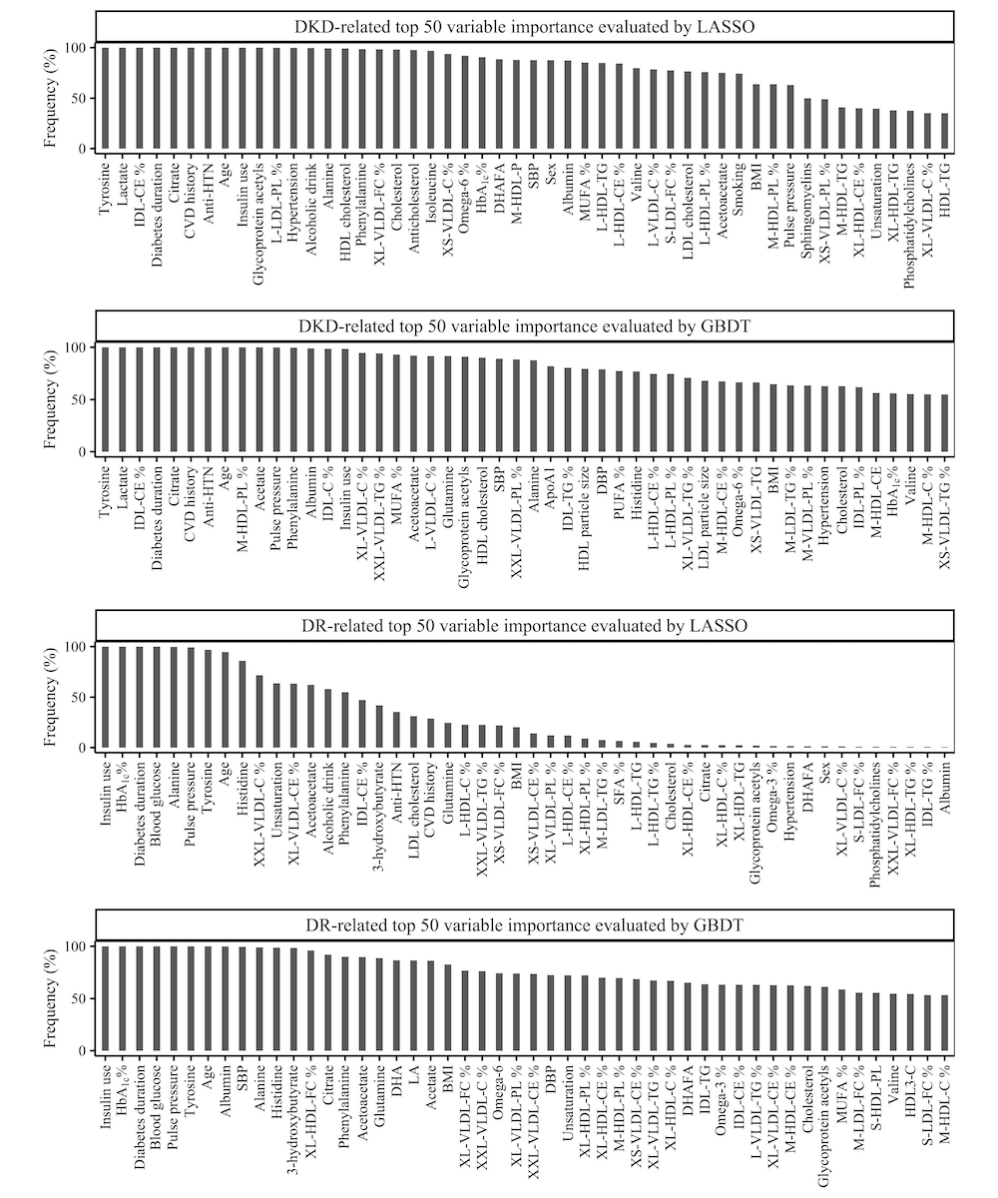
Top 50 machine-learned variables for DKD and DR. %: metabolites to total lipids ratio; anti-HTN: antihypertensive medication use; ApoA1: apolipoprotein A1; C: cholesterol; CE: cholesterol esters; CVD: cardiovascular disease; DBP: diastolic blood pressure; DKD: diabetic kidney disease; DHA: docosahexaenoic acid; DHAFA: antihypertensive medication use; DR: diabetic retinopathy; FC: free cholesterol; GBDT: gradient-boosting decision tree; HDL: high-density lipoprotein; HDL3: high-density lipoprotein 3; IDL: intermediate-density lipoprotein; L: large; LA: linoleic acid; LASSO: Least Absolute Shrinkage and Selection Operator; LDL: low-density lipoprotein; M: medium; MUFA: monounsaturated fatty acids; P: particles; PL: phospholipids; PUFA: polyunsaturated fatty acids; S: small; SBP: systolic blood pressure; SFA: saturated fatty acids; TG: triglycerides; VLDL: very-low-density lipoprotein; XL: very large; XS: very small; XXL: extremely large.

Figure 2 shows the top 50 variables arranged in descending orders of their variable importance. For DKD detection, LASSO identified 15 variables with a frequency exceeding the cutoff, while GBDT identified 13, so we used the top 15 for deriving the corresponding DKD models; then for DR, LASSO identified 10 variables, while GBDT identified 6, so we used the top 10 for DR.

**Figure 3:**
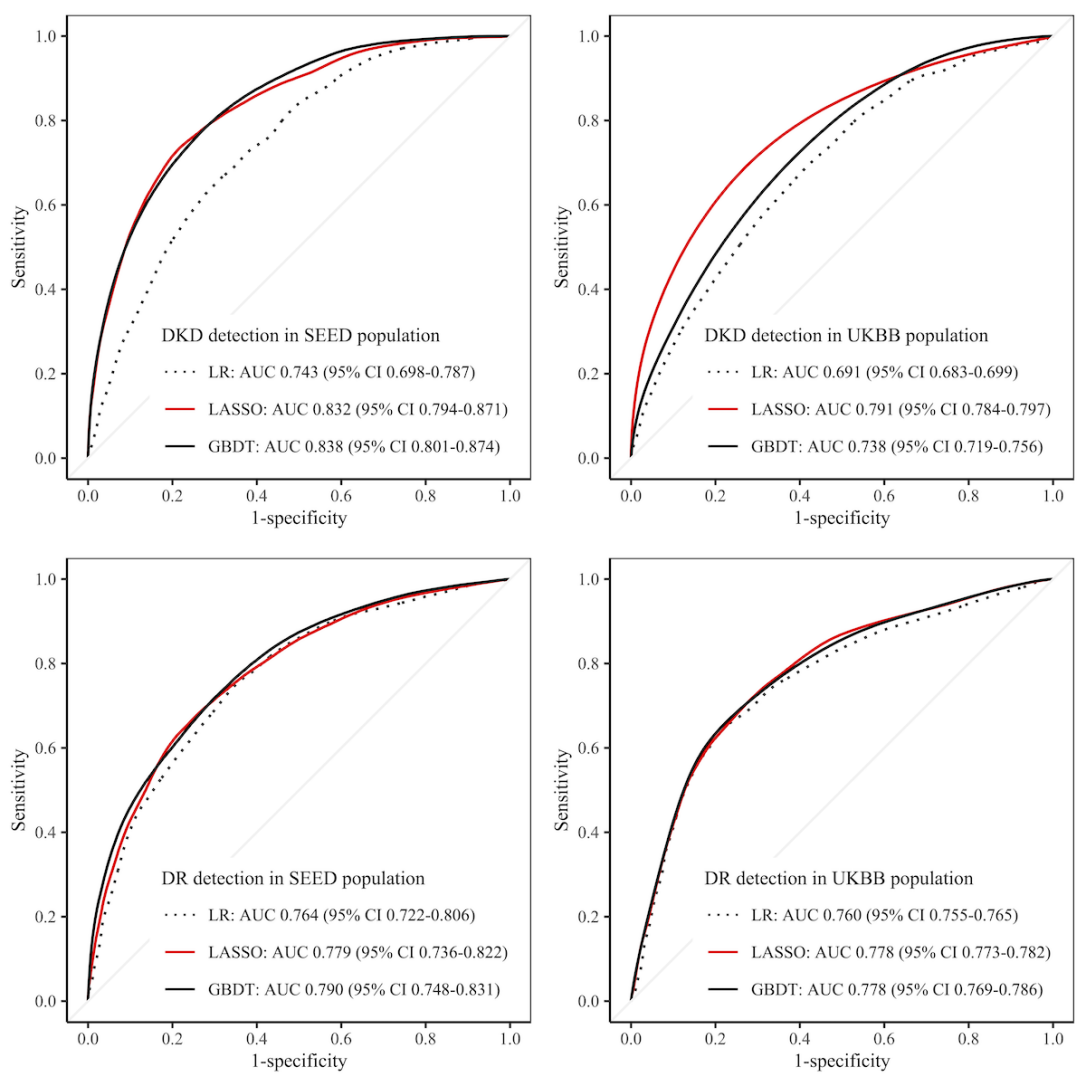
Receiver operating characteristic curves showing the model performances. AUC: area under the receiver operating characteristic curve; DKD: diabetic kidney disease; DR: diabetic retinopathy; GBDT: gradient-boosting decision tree; LASSO: Least Absolute Shrinkage and Selection Operator; LR: logistic regression; SEED: Singapore Epidemiology of Eye Diseases; UKBB: UK biobank.

In the internal validation, GBDT models performed the best with an AUC of 0.838 for DKD and 0.790 for DR, followed by LASSO with AUC values of 0.832 and 0.779, respectively. In contrast, LR only achieved AUC scores of 0.743 for DKD, and 0.764 for DR. In the external validation using UKBB data, LASSO models exhibited the best performance with AUC values of 0.791 for DKD, and 0.778 for DR. GBDT models achieved AUC scores of 0.738 and 0.778, respectively, while LR resulted in AUC values of 0.691 and 0.760 (Figure 3).

Further tests confirmed that the AUC scores of ML models were significantly higher than those obtained using traditional LR (2-sided *P*<.001). In terms of sensitivity and specificity at the optimal threshold (where sensitivity=specificity), LASSO and GBDT achieved comparable performance in internal validation (for DKD, 0.757 by LASSO vs 0.751 by GBDT; for DR, 0.708 by LASSO vs 0.709 by GBDT), and both were superior to LR (0.674 for DKD and 0.696 for DR). In external validation, LASSO performed the best, with 0.723 for DKD and 0.716 for DR. At 0.8 sensitivity, LASSO achieved 0.636 specificity for DKD detection in UKBB, and 0.617 for DR, outperforming the other 2 models (Table 1).

**Table 1:** Performance evaluation of machine-learned models.

Disease and data set		Algorithm		AUC^a^ (95% CI)		Optimal SN^b^/SP^c^		SP at SN=0.8		Sample size, n^d^	Cases, n (%)
**DKD^e^**											
	SEED^f^	LR^g^	0.743 (0.698-0.787)		0.674		0.54		2653		517 (19.5)
	SEED	LASSO^h^	0.832 (0.794-0.871)		0.757		0.703		2666		532 (20)
	SEED	GBDT^i^	0.838 (0.801-0.874)		0.751		0.709		2668		529 (19.8)
	UKBB^j^	LR	0.691 (0.683-0.699)		0.635		0.472		5236		345 (6.6)
	UKBB	LASSO	0.791 (0.784-0.797)		0.723		0.636		5090		333 (6.5)
	UKBB	GBDT	0.738 (0.719-0.756)		0.666		0.535		5543		366 (6.6)
**DR^k^**											
	SEED	LR	0.764 (0.722-0.806)		0.696		0.594		2597		653 (25.1)
	SEED	LASSO	0.779 (0.736-0.822)		0.708		0.596		2514		628 (25)
	SEED	GBDT	0.790 (0.748-0.831)		0.709		0.616		2598		655 (25.2)
	UKBB	LR	0.760 (0.755-0.765)		0.707		0.571		5492		336 (6.1)
	UKBB	LASSO	0.778 (0.773-0.782)		0.716		0.617		4678		280 (6)
	UKBB	GBDT	0.778 (0.769-0.786)		0.715		0.604		4833		296 (6.1)

^a^AUC: area under the receiver operating characteristic curve.

^b^SN: sensitivity.

^c^SP: specificity.

^d^Final sample size after variable selection and missing data removal.

^e^DKD: diabetic kidney disease.

^f^SEED: Singapore Epidemiology of Eye Diseases.

^g^LR: logistic regression.

^h^LASSO: Least Absolute Shrinkage and Selection Operator.

^i^GBDT: gradient-boosting decision tree.

^j^UKBB: UK biobank.

^k^DR: diabetic retinopathy.

In the sensitivity analysis, we evaluated the potential improvement in AUC by introducing additional variables in nested models (Multimedia Appendix 5). The results supported the effectiveness of using the top 10 variables for detecting DR in SEED. In line with this, using all 239 variables did not improve beyond what was achieved with the top 10 in both internal validation (LASSO 0.776 and GBDT 0.783) and external validation (LASSO 0.754 and GBDT 0.774). Yet for DKD, incorporating additional variables beyond the top 15 resulted in improved AUC in SEED. Specifically, using all 239 variables yielded higher AUC values of 0.859 for LASSO and 0.842 for GBDT. However, this increase may be attributed to overfitting, as a similar pattern was not observed in external validation. Further, using all available variables resulted in lower AUC values for both LASSO (0.694) and GBDT (0.721) in the UKBB cohort. Consequently, we decided to maintain the DKD model with the top 15 variables for its simplicity and effectiveness.

### Metabolites Selection

For DKD, both LASSO and GBDT selected 5 risk factors (duration of diabetes, history of CVD, antihypertensive medication use, age, and insulin use) and 4 metabolites (tyrosine, lactate, cholesterol esters to total lipid ratio in intermediate-density lipoprotein particles [IDL-CE%], and citrate). For any DR, both algorithms identified 6 risk factors (insulin use, HbA_1c_%, duration of diabetes, random blood glucose, age, and PP) and 2 metabolites (tyrosine and alanine). In the supplementary analysis, tyrosine was again selected for moderate or worse DR detection (Multimedia Appendix 6).

Table 2 shows the association of the machine-learned metabolites with DKD and DR in multivariable LR models. We found tyrosine to be negatively associated with both DKD (OR 0.65, 95% CI 0.58-0.73; *P*<.001) and DR (OR 0.90, 95% CI 0.81-1.00; *P*=.047). High levels of alanine were associated with increased DR prevalence (OR 1.31, 95% CI 1.18-1.45; *P*<.001), but with decreased DKD prevalence (OR 0.72, 95% CI 0.64-0.80; *P*<.001). Similarly, a high level of lactate was linked to higher DR prevalence (OR 1.16, 95% CI 1.05-1.28; *P*=.004) but with lower DKD prevalence (OR 0.71, 95% CI 0.63-0.80; *P*<.001). Finally, a high level of citrate was associated with increased DKD prevalence (OR 1.90, 95% CI 1.70-2.12; *P*<.001), while high IDL-CE% was linked to decreased DKD prevalence (OR 0.46, 95% CI 0.40-0.53; *P*<.001).

**Table 2:** Association of selected metabolites with DKD^a^ and DR^b^.

Metabolite	DKD detection		DR detection	
	OR^c^ (95% CI)	*P* value	OR (95% CI)	*P* value
Alanine	0.72 (0.64-0.80)	<.001	1.31 (1.18-1.45)	<.001
Tyrosine	0.65 (0.58-0.73)	<.001	0.90 (0.81-1.00)	.047
Citrate	1.90 (1.70-2.12)	<.001	0.93 (0.84-1.03)	.2
IDL-CE%^d^	0.46 (0.40-0.53)	<.001	0.89 (0.79-1.01)	.1
Lactate	0.71 (0.63-0.80)	<.001	1.16 (1.05-1.28)	.004

^a^DKD: diabetic kidney disease.

^b^DR: diabetic retinopathy.

^c^OR: odds ratio.

^d^IDL-CE%: cholesterol esters to total lipid ratio in intermediate-density lipoprotein particles.

## Discussion

### Principal Findings

ML selected age, use of insulin, duration of diabetes, and circulating tyrosine as the most important markers for DKD and DR detection in the SEED population with diabetes. Additionally, DKD was associated with the use of antihypertensive medications, CVD history, and 3 metabolites (lactate, citrate, and IDL-CE%), whereas DR was additionally linked to HbA_1c_, random blood glucose, PP, and alanine.

The ML models developed in the SEED cohort with diabetes were externally validated using UKBB data. In both cohorts, ML models outperformed the traditional LR in terms of AUC, sensitivity, and specificity, demonstrating their potential to discover novel biomarkers and enable disease screening when integrated with health care and metabolite data.

### Comparison With Prior Work

Our main data set included a comprehensive set of 19 risk factors and 220 circulating metabolites measured in 2772 individuals. The detailed patient profiling with a robust sample size allowed an opportunity to identify the markers most relevant to DKD and DR, offering insights into the systematic alteration of metabolism and underlying pathways.

In line with the previous literature [4,13], ML consistently identified 3 key factors—diabetes duration, age, and the use of insulin—as the top risk factors for both DKD and DR. However, ML also revealed novel aspects concerning some established risk factors for these conditions. For instance, our ML models exhibited a notable preference for PP over SBP and DBP in DR detection, supporting the study by Yamamoto et al [21] that PP, as a surrogate marker of arterial stiffness, reflected both the SBP elevation and DBP reduction, thereby carrying more predictive information for DR than other blood pressure metrics. Another example was assessing the relative importance of glycemia control indicators, where HbA_1c_% consistently received a higher selection frequency in our ML models compared to random blood glucose levels. This was probably because HbA_1c_% was averaged to reflect a mean shift with much less random noise, while random blood glucose data might carry more noise from life cycle changes and interindividual variability [22]. Interestingly, some well-established risk factors usually included such as sex did not appear in the top-ranking lists by ML, although this variable had been selected by traditional LR models on the same population in previous studies [23]. This could be because sex is an intrinsic component of other phenotypes. For instance, male sex was associated with CVD [24], a condition well-known to be linked to DKD and DR [2].

We noted high levels of tyrosine, an aromatic amino acid, to be negatively associated with the prevalence of DKD and DR, supporting the ADVANCE trial where increased tyrosine concentration was linked to a decreased risk of diabetic microvascular events (hazard ratio 0.78, 95% CI 0.67-0.91) [12]. Tyrosine is mainly synthesized from phenylalanine hydroxylation in the liver and kidney; impaired kidney function is therefore associated with reduced phenylalanine hydroxylase activity characterized by low blood tyrosine levels [12]. Additionally, tyrosine serves as a precursor to catecholamine neurotransmitters (dopamine, norepinephrine, and epinephrine), and plays a pivotal role in central nervous system functions and activities [25]. In metabolic disorders such as diabetes, reduced blood tyrosine can affect its uptake into the brain and also the synthesis and release of transmitters, thereby altering hormonal function, affective state, and blood pressure [25], potentially linked to higher microvascular risks.

Another amino acid selected by ML was alanine, and its higher concentration in blood was associated with higher DR prevalence, but lower DKD prevalence. In the ADVANCE trial, a negative association was reported between circulating alanine and an aggregated microvascular outcome, defined as new or worsening nephropathy or retinopathy (hazard ratio 0.86, 95% CI 0.76-0.98) [12]. In other research, high levels of blood amino acids such as alanine have been linked to inhibited insulin signaling to glucose transport, phosphorylation, and glycogen synthesis, causing insulin resistance, which impairs hepatic mitochondrial function in patients with diabetes and contributes to diabetes microvascular complications such as DR [26,27].

Among the ML-selected metabolites for DKD, high levels of lactate were found to be associated with lower DKD prevalence. However, another study conducted in the DKD population suggested an accumulation of acid due to abnormal lactate metabolism causing fibrosis and mitochondrial abnormalities, leading to further kidney damage [28]. Nevertheless, further investigations are required to understand the complex association between circulating lactate and DKD. Another metabolite identified was citrate, which showed a positive association with DKD in SEED. In people with DKD, an elevated plasma citrate level has been linked to reduced organic anion transport and dysregulated mitochondrial functions of the kidney tissues [8]. Our study also highlighted the significance of IDL-CE% in diabetic microvascular complications, revealing its inverse association with prevalent DKD in the SEED population with diabetes. Aberrations in lipoprotein composition have been reported to be indicative of insulin resistance and impaired glucose tolerance in the general population, known to cause future diabetes [9,27]. In people with type 1 DKD, abnormality in lipoproteins clearance was further linked to impaired kidney function [29].

For biomarker discovery, traditional studies often relied on LR models to examine metabolites one by one separately [12,30], with stringent model assumptions and multiple testing corrections [9,31]. Herein, our ML approach was more efficient in the sense that it simultaneously examines all variables for potential associations. While LASSO was limited to detecting linear associations, we implemented GBDT as a complementary model to additionally assess nonlinear terms and high-order interactions. As was shown in Figure 2, DR-related variables in GBDT had higher selection frequencies than in LASSO, suggesting the existence of such complex associations. Still, LASSO models achieved superior performance in external validation, indicating a prominent contribution of linear associations to DKD and DR detection. Another highlight of our methodology was the repeated cross-validation, which reduced the influence of potential outliers and ensured the randomness of sampling, thereby generating results more robust than those relying on a fixed training set. This approach also allowed us to quantify individual contributions of variables based on their selection frequencies. However, alternative definitions of variable importance scores [32] may be considered in a future study to potentially refine the variable selection process.

Globally, three-quarters of those with diabetes live in low- and middle-income countries, particularly India and China [33]. While the risk profile of Asian patients with diabetes differs from that in high-income “Western” societies in terms of age, BMI, lifestyle, diet, and many other aspects [33], there has been limited discussion on Asian populations with diabetes. Our study, conducted in Asian populations (Malay, Indian, and Chinese), may contribute to the topic by allowing an opportunity to identify the commonalities and differences between DKD and DR in terms of circulating metabolic traits, offering insights into the systematic alteration of metabolism in diabetes.

### Limitations

In total, 1 limitation of this study was that we did not separate study subjects by diabetes type. Since around 95% of the SEED population with diabetes had type 2 diabetes, our results would mainly reflect the variable associations with type 2 diabetes. While these associations hold potential for hypothesis generation and disease detection, the cross-sectional nature of our study highlights the need for caution in drawing causal inferences. Future longitudinal studies are warranted to establish temporal associations based on our current findings.

Another issue was data availability—albuminuria, an important indicator of kidney disease [13], and 3 metabolites (pyruvate, glycerol, and glutamine) were missing in Malay individuals of the SEED population. Hence, these variables were excluded from the analysis. Certain diabetic medications were reported to potentially interfere with circulating metabolite levels [12,34,35]. However, we could only account for the use of insulin due to limited data availability on UKBB medication profiles. Furthermore, we noted a difference in defining DR between our development data set (SEED), which used the standard ETDRS classification system, and the external test set (UKBB), where DR cases were identified using ICD-10 codes. This variance in outcome definition might have introduced some degree of misclassification in UKBB. Additionally, due to the absence of ETDRS-based severity scores in UKBB, validation of the supplementary model for moderate or worse DR in supplementary analysis was not feasible. Nevertheless, it was noteworthy that the features identified in SEED remained relevant for enhancing DR detection in the UKBB data set. However, we acknowledge that the discrepancy in DR definition underscores the need for caution when interpreting results, particularly in the context of clinical or practical applications. Future research may benefit from adopting a uniform approach to DR classification to minimize such limitations and enhance result validity.

### Conclusions

In conclusion, current ML models developed using the SEED population with diabetes and subsequently validated in UKBB showed superior performance compared to traditional LR for DKD and DR detection. ML highlighted age, use of insulin, diabetes duration, and tyrosine as the most influential factors in DKD and DR. Additionally, DKD was associated with high levels of citrate, low levels of lactate, and low IDL-CE%; while DR was linked to higher levels of alanine. The integration of ML with health care data and metabolomics could facilitate biomarker selection and enable disease screening.

## Appendix

Multimedia Appendix 1 List of 239 Singapore Epidemiology of Eye Diseases (SEED) variables included for machine learning feature selection.

Multimedia Appendix 2 Distribution of missing data in Singapore Epidemiology of Eye Diseases (SEED), column-wise and row-wise histograms.

Multimedia Appendix 3 Population characteristics of Singapore Epidemiology of Eye Diseases (SEED) stratified by diabetic kidney disease (DKD) and diabetic retinopathy (DR) status.

Multimedia Appendix 4 Comparative analysis of population characteristics: Singapore Epidemiology of Eye Diseases (SEED) vs UK biobank.

Multimedia Appendix 5 Area under the receiver operating characteristic curve performance of nested models in internal validation with varying numbers of machine-learned variables.

Multimedia Appendix 6 Top-50 machine-learned variables for moderate or worse diabetic retinopathy (DR) detection.

## Abbreviations

AUC: area under the receiver operating characteristic curve
CVD: cardiovascular disease
DBP: diastolic blood pressure
DKD: diabetic kidney disease
DR: diabetic retinopathy
eGFR: estimated glomerular filtration rate
ETDRS: Early Treatment Diabetic Retinopathy Study
GBDT: gradient-boosting decision tree
HbA1c: hemoglobin A1c
HDL: high-density lipoprotein
ICD-10: International Classification of Diseases, Tenth Revision
IDL-CE%: cholesterol esters to total lipid ratio in intermediate-density lipoprotein particles
LASSO: Least Absolute Shrinkage and Selection Operator
LR: logistic regression
ML: machine learning
OR: odds ratio
PP: pulse pressure
SBP: systolic blood pressure
SEED: Singapore Epidemiology of Eye Diseases
UKBB: UK biobank

## Data Availability

As the Singapore Epidemiology of Eye Diseases (SEED) study involves human participants, the data cannot be made freely available in this paper, the supplemental files, or a public repository due to ethical restrictions. Nevertheless, the data are available from the Singapore Eye Research Institutional Ethics Committee for researchers who meet the criteria for access to confidential data. Interested researchers can send data access requests to the Singapore Eye Research Institute. The UK biobank (UKBB) test data set was obtained from the UKBB (application 45925). Data cannot be shared publicly due to the violation of patient privacy and the absence of informed consent for data sharing.
