# Supplementary material for: Development and External Validation of Machine Learning Models for Diabetic Microvascular Complications: Cross-Sectional Study With Metabolites
Source: J Med Internet Res. 2024 Mar 28;26:e41065. doi: 10.2196/41065 (PMC11009843; doi:10.2196/41065)

Distribution of missing data in 239 variables of SEED

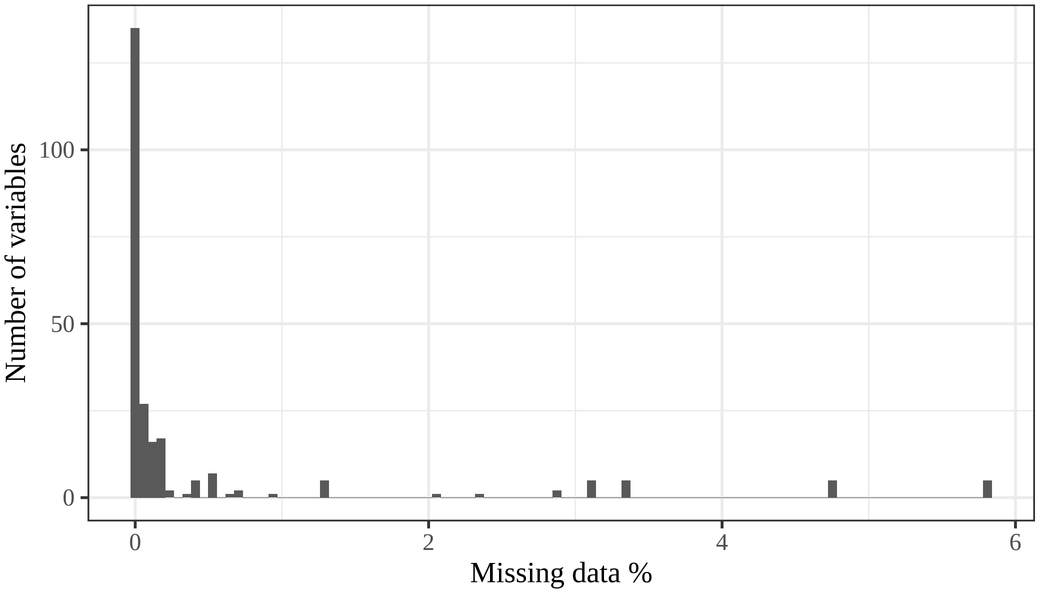

Distribution of missing data in 2,772 participants of SEED

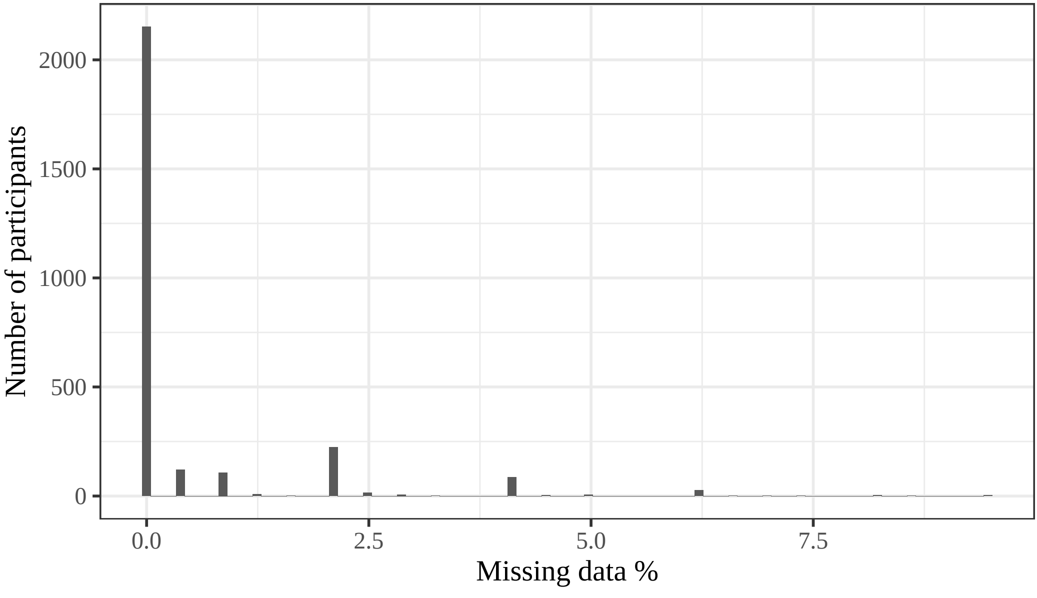

Supplement: Multimedia Appendix 2 [file jmir_v26i1e41065_app2.pdf]
