# Supplementary material for: Development and External Validation of Machine Learning Models for Diabetic Microvascular Complications: Cross-Sectional Study With Metabolites
Source: J Med Internet Res. 2024 Mar 28;26:e41065. doi: 10.2196/41065 (PMC11009843; doi:10.2196/41065)
Supplement: Multimedia Appendix 3 [file jmir_v26i1e41065_app3.pdf]

	No DKD and no DR (n=1657)	DKD-alone (n=338)	DR-alone (n=496)	DKD and DR (n=183)	***P***
Age, median [IQR], y	59.2 [52.0, 67.4]	69.6 [62.6, 74.4]	59.5 [51.9, 65.0]	66.1 [61.0, 72.3]	<.001
Female, n (%)	825 (49.8)	157 (46.4)	228 (46.0)	102 (55.7)	.09
Current smoker, n (%)	253 (15.3)	34 (10.1)	72 (14.5)	11 (6.0)	.001
Alcohol consumption, n (%)	126 (7.6)	11 (3.3)	61 (12.4)	3 (1.6)	<.001
Anti-diabetic medication use, n (%)	855 (51.6)	200 (59.2)	387 (78.0)	149 (81.4)	<.001
Duration of diabetes, median [IQR], y	2.0 [0.0, 6.7]	4.2 [0.0, 10.8]	10.3 [3.6, 17.8]	13.4 [7.0, 21.0]	<.001
Insulin use, n (%)	28 (1.7)	14 (4.1)	61 (12.3)	34 (18.6)	<.001
Glycated hemoglobin, mean (SD), %	7.6 (1.6)	7.4 (1.6)	8.3 (1.9)	8.2 (1.8)	<.001
Random blood glucose, mean (SD), mmol/L	9.3 (4.3)	9.1 (4.3)	11.4 (5.8)	11.3 (5.8)	<.001
History of cardiovascular disease, n (%)	242 (14.6)	99 (29.3)	95 (19.2)	63 (34.4)	<.001
Hypertension, n (%)	1244 (75.3)	317 (94.1)	400 (80.8)	177 (96.7)	<.001
Antihypertensive medication use, n (%) [a]	773 (62.1)	237 (74.8)	268 (67.0)	138 (78.0)	<.001
Pulse pressure, mean (SD), mm Hg	64.0 (16.7)	72.8 (18.6)	67.4 (17.5)	79.8 (19.4)	<.001
Systolic blood pressure, mean (SD), mm Hg	142.7 (20.5)	150.7 (23.7)	145.4 (21.6)	156.4 (26.2)	<.001
Diastolic blood pressure, mean (SD), mm Hg	78.7 (10.3)	77.8 (10.8)	78.0 (10.3)	76.6 (11.6)	.009
Anti-cholesterol medication use, n (%)	652 (40.1)	170 (51.2)	217 (45.0)	98 (55.1)	<0.001

Serum total cholesterol, mean (SD), mmol/L	5.2 (1.2)	5.2 (1.3)	5.0 (1.3)	5.1 (1.5)	<0.001
Serum high-density lipoprotein cholesterol, mean (SD), mmol/L	1.1 (0.3)	1.2 (0.3)	1.1 (0.3)	1.2 (0.4)	.04
Body mass index, mean (SD), Kg/m^2	27.1 (4.8)	27.1 (4.7)	26.2 (4.5)	26.9 (5.5)	<0.001
Estimated glomerular filtration rate, mean (SD), $mL/min/1.73m^2$	87.6 (14.8)	45.8 (11.8)	88.6 (17.4)	41.2 (14.7)	<0.001

Abbreviations: SD, standard deviation; IQR, interquartile range.

Total sample size was 2674 excluding 79 participants missing DR status and 19 missing eGFR results.

[a] Among those with hypertension
