# Supplementary material for: Development and External Validation of Machine Learning Models for Diabetic Microvascular Complications: Cross-Sectional Study With Metabolites
Source: J Med Internet Res. 2024 Mar 28;26:e41065. doi: 10.2196/41065 (PMC11009843; doi:10.2196/41065)
Supplement: Multimedia Appendix 4 [file jmir_v26i1e41065_app4.pdf]

	**SEED (*n*=2,772)**	**UK Biobank (*n*=5,843)**	***P***
Age, median [IQR], y	61.7 [53.5, 69.4]	61.0 [55.0, 65.0]	<.001
Female, n (%)	1,361 (49.1)	2,090 (35.8)	<.001
Ethnicity	Malay, Indian, Chinese	British, etc.	<.001
Current smoker, n (%)	377 (13.6)	3,193 (54.6)	<.001
Alcohol consumption, n (%)	204 (7.4)	5,342 (91.4)	<.001
Duration of diabetes, median [IQR], y	3.9 [0.0, 10.7]	5.0 [2.0, 10.0]	<.001
Insulin use, n (%)	143 (5.2)	1,245 (21.3)	<.001
Glycated hemoglobin, mean (SD), %	7.7 (1.7)	7.0 (1.3)	<.001
Random blood glucose, mean (SD), mmol/L	9.8 (4.8)	7.6 (3.4)	<.001
History of cardiovascular disease, n (%)	519 (18.7)	245 (4.2)	<.001
Hypertension, n (%)	2,228 (80.5)	4,834 (82.8)	.01
Antihypertensive medication use, n (%) [a]	1,474 (66.2)	3,727 (89.5)	<.001
Pulse pressure, mean (SD), mm Hg	67.1 (18.0)	74.3 (13.3)	<.001
Systolic blood pressure, mean (SD), mm Hg	145.5 (22.2)	142.9 (18.2)	<.001
Diastolic blood pressure, mean (SD), mm Hg	78.3 (10.5)	81.2 (10.4)	<.001
Anti-cholesterol medication use, n (%)	1,183 (43.6)	4,479 (76.7)	<.001
Body mass index, mean (SD), Kg/m^2	26.9 (4.8)	31.3 (5.7)	<.001
Serum total cholesterol, mean (SD), mmol/L	5.2 (1.2)	4.5 (1.0)	<.001
Serum high-density lipoprotein cholesterol, mean (SD), mmol/L	1.1 (0.3)	1.2 (0.3)	<.001
Any diabetic retinopathy, n (%)	685 (25.4)	355 (6.1)	<.001
Diabetic kidney disease, n (%)	555 (20.2)	374 (6.7)	<.001
Estimated glomerular filtration rate, mean (SD), $mL/min/1.73m^2$	78.9 (23.0)	88.6 (17.1)	<.001

Abbreviations: SD, standard deviation; IQR, interquartile range.

Subgroups may not add up due to the presence of missing data.

[a] Among those with hypertension
