# Supplementary figures and images for: Development and External Validation of Machine Learning Models for Diabetic Microvascular Complications: Cross-Sectional Study With Metabolites
Source: J Med Internet Res. 2024 Mar 28;26:e41065. doi: 10.2196/41065 (PMC11009843; doi:10.2196/41065)

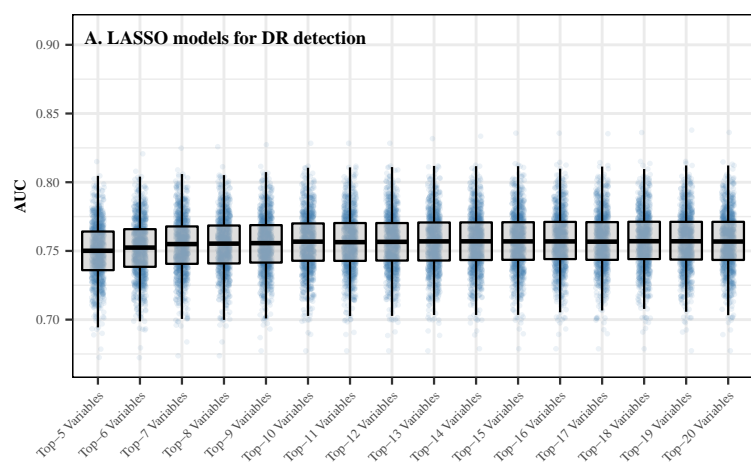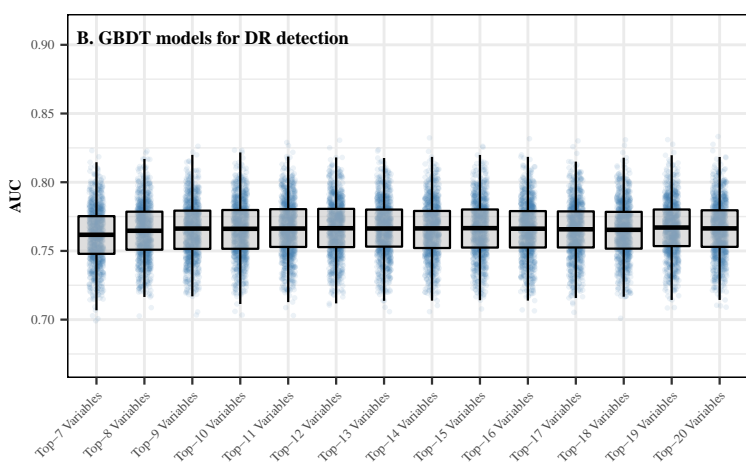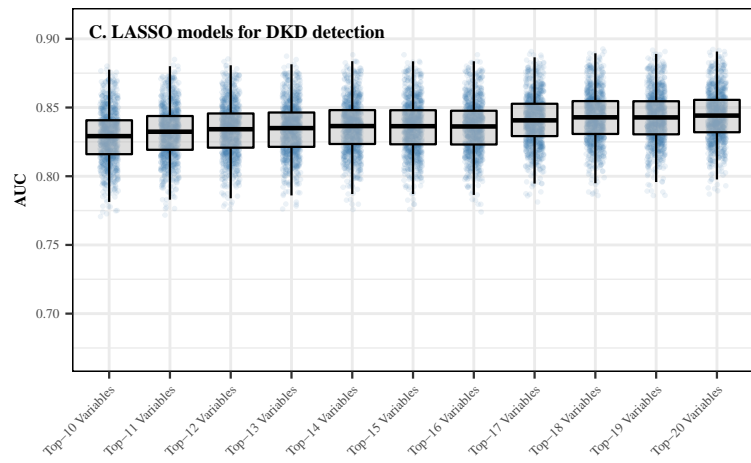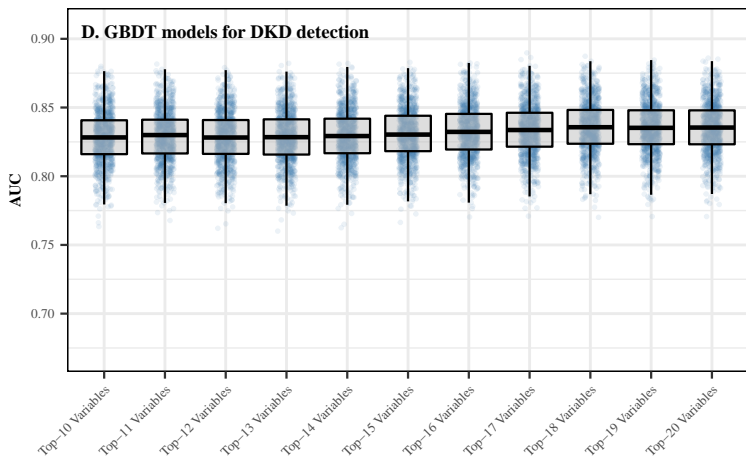

Supplement: Multimedia Appendix 5 [file jmir_v26i1e41065_app5.pdf]

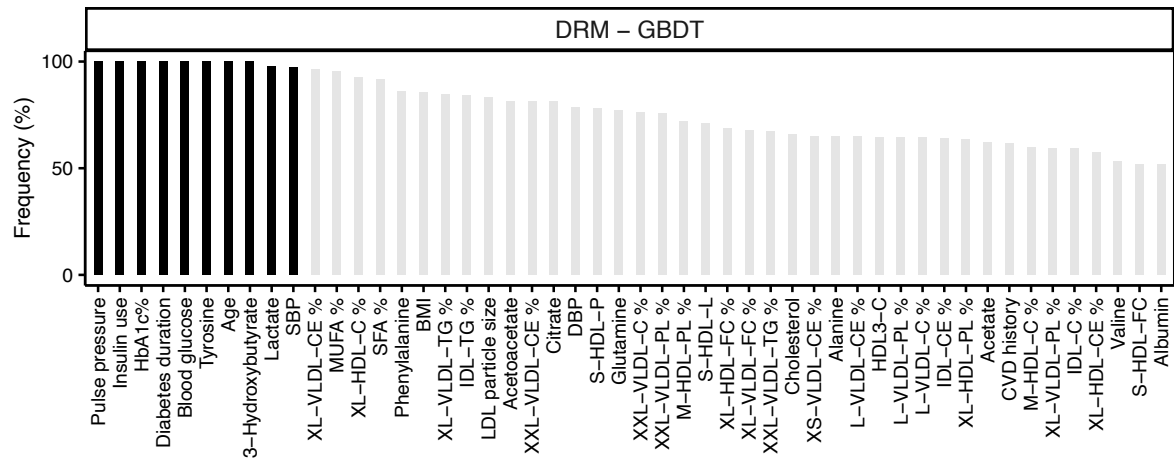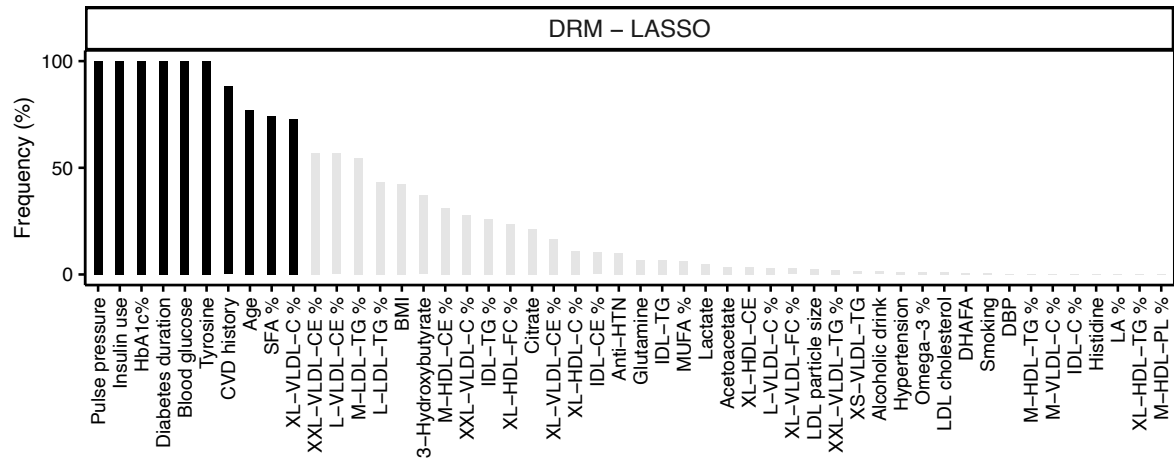

Supplement: Multimedia Appendix 6 [file jmir_v26i1e41065_app6.pdf]
